# Identification of reference genes for the normalization of retinal mRNA expression by RT-qPCR in oxygen induced retinopathy, anemia, and erythropoietin administration

**DOI:** 10.1371/journal.pone.0284764

**Published:** 2023-04-25

**Authors:** Mandkhai Molomjamts, Ellen C. Ingolfsland

**Affiliations:** Department of Pediatrics, Division of Neonatology, University of Minnesota, Minneapolis, MN, United States of America; Children’s Hospital Boston, UNITED STATES

## Abstract

**Background:**

Anemia and retinopathy of prematurity (ROP) are common comorbidities experienced by preterm infants, yet the role of anemia on the pathogenesis of ROP remains unclear. Reverse-transcriptase quantitative polymerase chain reaction (RT-qPCR) is a sensitive technique for estimating the gene expression changes at the transcript level but requires identification of stably expressed reference genes for accurate data interpretation. This is particularly important for oxygen induced retinopathy studies given that some commonly used reference genes are sensitive to oxygen. This study aimed to identify stably expressed reference genes among eight commonly used reference genes in the neonatal rat pups’ retina upon exposure to cyclic hyperoxia-hypoxia, anemia, and erythropoietin administration at two age groups (P14.5 and P20) using Bestkeeper, geNorm, and Normfinder, three publicly available, free algorithms, and comparing their results to the in-silico prediction program, RefFinder.

**Results:**

The most stable reference gene across both developmental stages was Rpp30, as predicted by Genorm, Bestkeeper, and Normfinder. RefFinder predicted Tbp to be the most stable across both developmental stages. At P14.5, stability varied by prediction program; at P20, RPP30 and MAPK1 were the most stable reference genes. Gapdh, 18S, Rplp0, and HPRT were predicted as the least stable reference genes by at least one of the prediction algorithms.

**Conclusion:**

Expression of Rpp30 is the least affected by experimental conditions of oxygen induced retinopathy, phlebotomy induced anemia and erythropoietin administration at both timepoints of P14.5 and P20.

## 1. Introduction

Iron deficiency is the most common micronutrient deficiency in the world, and it is experienced by 25%-85% of preterm infants [1–4]. Iron deficiency puts preterm infants at risk for disrupted neurodevelopment, neuronal differentiation and myelination and leads to iron deficiency anemia [5]. This preexisting anemia of prematurity is often exacerbated by phlebotomy induced anemia (PIA) which is caused by the frequent need for blood analysis while in the neonatal intensive care unit (NICU). Anemia may also increase the risk for retinopathy of prematurity (ROP), a common blinding disease that accounts up to 40% childhood blindness worldwide [6,7]. Supplemental use of oxygen in the NICU disrupts retinal neurovascular growth and leads to ROP. The retina is the most metabolically active tissue in the body and consists of diverse neuronal types and consumes oxygen at rates higher than neurons in the brain [8].

Thus, the impact of iron deficiency and depletion of oxygen supply caused by anemia on the pathogenesis of ROP must be defined for enhanced treatment. It has been difficult to isolate the impact of anemia in the clinical setting given the effect of blood transfusions and other underlying factors.

Quantitative analysis of retinal mRNA by real-time quantitative polymerase chain reaction (RT-qPCR) is a powerful tool for investigating the impact of experimental conditions on the developing retina. Oxygen-induced retinopathy preclinical models (OIR) are commonly used to study altered retinal angiogenesis and neovascularization and are pertinent to the study of ischemic retinopathies such as retinopathy of prematurity and diabetic retinopathy [9]. The rat retina vascularizes postnatally in a pattern that mimics human retinal development, vascularizing centrally (from the optic nerve) to peripherally (to the outer edge of the retina). In this model, the rat OIR model alters retinal vascularization during phases of rapid retinal growth and development, and study during both the avascular and neovascular phases of retinopathy provide distinct insights. Addition of other experimental conditions during this time period are likewise highly time sensitive. Furthermore, the retina is a complex tissue, comprised of more than 60 cell types that have morphologic and functional differences during development [10]. Quantitative determinations of retinal development therefore require care to differentiate time point and cell type being studied.

RT-qPCR allows investigation into the complex mechanisms underlying retinal development with high sensitivity and specificity. RT-qPCR accuracy, however, depends heavily on the selected reference gene stability. Parallel quantification of reference genes as endogenous controls is the optimal way to correct inevitable experimental variations in addition to the calculation of true relative quantification (fold change) of genes. No single gene is found to be expressed uniformly across tissues or within cells under different conditions. For instance, commonly used GAPDH is shown to be sensitive in hypoxic conditions and transcriptionally modulated [11]. This highlights the importance of reference gene validation according to the experimental conditions.

Currently, there is no consensus regarding reference gene selection for RT-qPCR analysis of neonatal rat retina tissue across the experimental conditions of OIR, anemia, its treatment with recombinant human erythropoietin (EPO) and room air control (RA).

Therefore, the objective of this study was to measure the stability of 8 commonly used reference genes in RT-qPCR of whole retinal lysates at two developmentally relevant time points across these 4 experimental conditions. Use of a single reference gene may not be sufficient in RT-qPCR data normalization, but this study offers a set of reference genes from the stability ranking. The stability of reference genes was evaluated by Bestkeeper, geNorm, and Normfinder algorithms individually in addition to the *in silico* prediction program, *RefFinder*, which integrates previously mentioned algorithms and comparative delta Ct algorithm. We subsequently demonstrated the significance of validating reference gene selection by quantifying target gene expression levels normalized by several candidate genes.

## 2. Results

### 2.1 Analysis of reference gene stability

Efficiency of RT-qPCR for all tested probes ranged within the acceptable range of 90–110% (Table 1) [12].

**Table 1:** Reference gene candidates evaluated by this study and their qPCR amplification efficiency.

Gene symbol	Gene name	GenBank Accession number	Supplier Assay ID (Taqman probe)	Function	qPCR Efficiency (%)
**Alox-15**	Arachidonate 15-lipoxygenase	NM_031010.2	Rn00696151_m1	Lipoxygenase enzyme	102.1
**GAPDH**	Glycealdehyde-3-phosphate dehydrogenase	NM_017008.4	Rn01775763_g1	Oxidoreductase in glycolysis and gluconeogenesis	94.4
**HPRT**	Hypoxanthine phosphoribosyl transferase	NM_012583.2	Rn01527840_m1	Metabolic salvage of purines	91
**MAPK1**	Mitogen-Activated Protein Kinase 1	NM_053842.2	Rn00671828_m1	Signal transduction	98.4
**PPIA**	Peptidylprolyl Isomerase A	NM_017101.1	Rn00690933_m1	Chaperone protein	98.0
**RPP30**	Ribonuclease P/MRP Subunit P30	NP_001178012	Rn01479850_m1	RNA polymerase III transcription factor	96.7
**Rplp0**	Ribosomal protein, large, P0	NM_022402.2	Rn03302271_gH	Structural component of ribosomal subunit	96.7
**18S**	Ribosomal protein 18S	NM_213557.1	Rn01428913_gH	Ribosome subunit	95.28
**TBP**	TATA box binding protein	NM_001004198.1	Rn01455646_m1	RNA polymerase II transcription factor	98.0

### 2.2 Bestkeeper analysis

**Fig 1:**
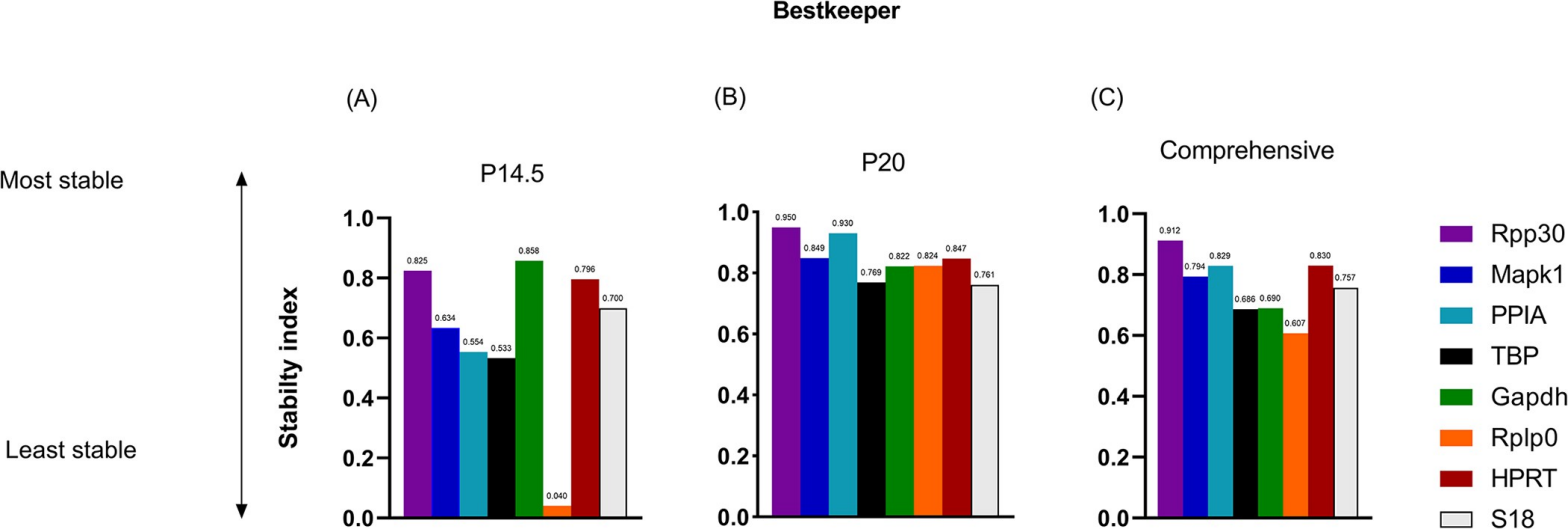
Stability ranking of 8 candidate reference genes by *Bestkeeper*. Higher stability index is associated with higher stability. (A) Early, highly avascular time point, P14.5. (B) Late time point where neovascularization is peaking, P20. (C) Comprehensive ranking of reference genes across both time points of P14.5.

All reference genes were evaluated by Bestkeeper algorithm, and the S.I. was expressed in terms of r-value, coefficient of correlation. High stability of a candidate gene is correlated to a higher r-value [13]. Fig 1A displays the stability ranking at P14.5, Fig 1B displays the stability ranking at P20, and Fig 1C displays the comprehensive reference gene stability across both time points.

Across all groups and time points, RPP30 was the most stable reference gene (S.I. 0.912) followed by HPRT (S.I. 0.83) and PPIA (S.I. 0.829) (Fig 1C). At P14.5, GAPDH (S.I. 1.000) and RPP30 (S.I. 0.825) were the most stable reference genes (Fig 1A). At P20, RPP30 (S.I. 0.950) and PPIA (S.I. 0.930) were the most stable genes (Fig 1B).

The least stable genes at P20 and across the comprehensive ranking of all groups and time points were Rplp0 (S.I. 0.607) and TBP (S.I. 0.686), respectively. At P14.5, Rplp0 (S.I. 0.04) and TBP (S.I.0.533) were the least stable reference genes.

### 2.3 Normfinder analysis

**Fig 2:**
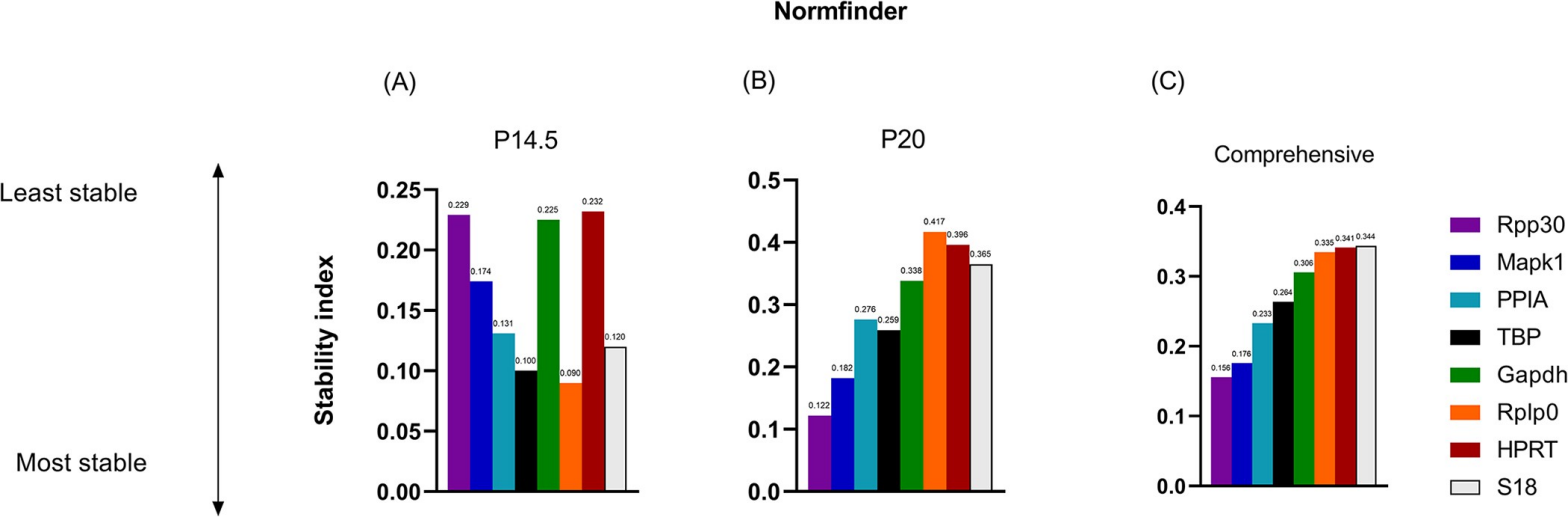
Stability ranking of 8 candidate reference genes by *Normfinder*. Lower stability index is associated with higher stability. (A) Early, highly avascular time point, P14.5. (B) Late time point where neovascularization is peaking, P20. (C) Comprehensive ranking of reference genes across both time points of P14.5.

The Normfinder algorithm was used to rank the stability of candidate reference genes. The stability index of Normfinder is calculated as a stable value for each gene, and a lower value indicates more stability [14]. Normfinder also identifies the best combination of two genes. RPP30 (S.I. 0.156) was the most stable reference gene, followed by MAPK1 (S.I. 0.176) for the comprehensive ranking of both timepoints (Fig 2C). The best combination of two genes across both timepoints was 18S and Tbp (S.I. 0.130). At P14.5, Rplp0 (S.I. 0.09) and TBP (S.I. 0.1) were the most stable reference genes, and the best combination of two genes was 18S and Tbp (S.I. 0.061). At P20, RPP30 (S.I. 0.122) and MAPK1 (S.I. 0.182) were the most stable reference genes (Fig 2A and 2B), and the best combination of two genes was Mapk1 and Rpp30 (S.I. 0.135). HPRT (S.I. 0.232), Rplp0 (S.I. 0.417), and 18S (S.I. 0.344) were the least stable genes at P14.5, P20, and at the comprehensive rankings, respectively.

### 2.4 GeNorm analysis

**Fig 3:**
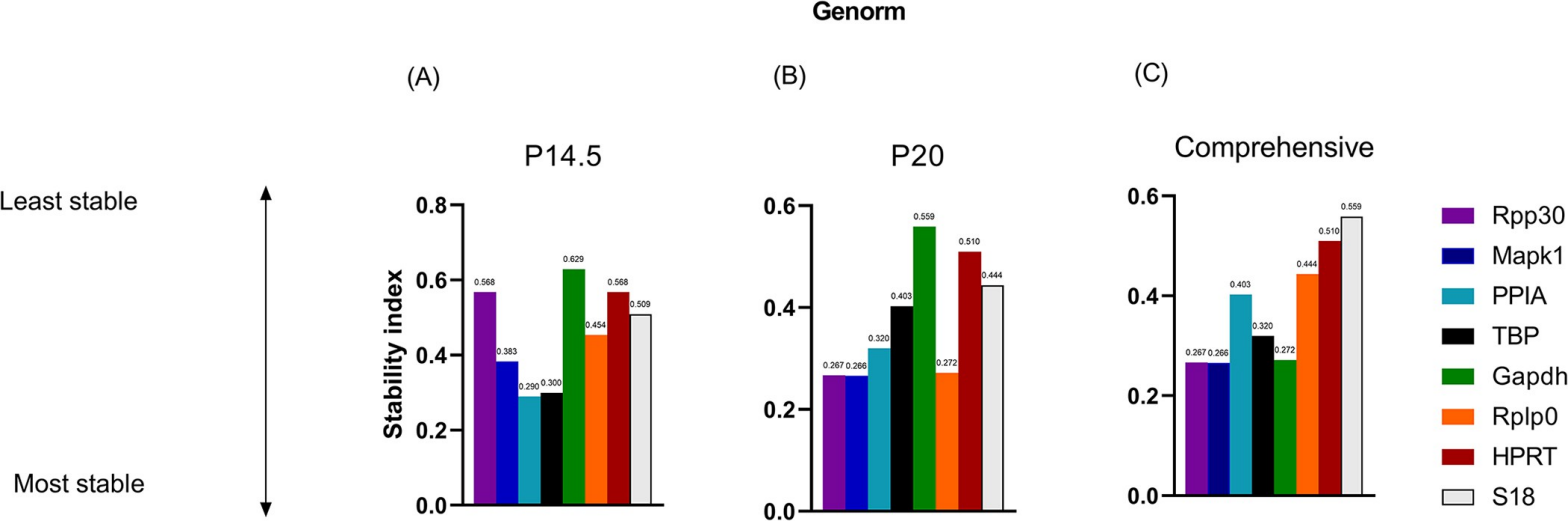
Stability ranking of 8 candidate reference genes by *geNorm*. Lower stability index is associated with higher stability. (A) Early, highly avascular time point, P14.5. (B) Late time point where neovascularization is peaking, P20. (C) Comprehensive ranking of reference genes across both time points of P14.5.

All reference genes were analyzed by geNorm algorithm, which calculated by the M-value for each gene as the arithmetic mean of all pairwise variation [15]. A lower M-value indicates higher stability of candidate reference genes. PPIA (S.I. 0.290) was the most stable reference gene at P14.5, while MAPK1 (S.I. 0.266) was the most stable reference gene at P20 (Fig 3A and 3B). MAPK1 (S.I. 0.266) and RPP30 (S.I. 0.267) were the most stable reference genes in the comprehensive ranking list (Fig 3C). GAPDH (S.I. 0.629 and 0.559) was the least stable reference gene at both P14.5 and P20. 18S (S.I. 0.559) was the least stable reference gene in the comprehensive ranking.

V_n_/V_n+1_ calculations of sequential numbers of reference genes were performed for each individual time point and comprehensively across both time points to determine the appropriate number of reference genes necessary for normalization. In each of these analyses, V_2_/V_3_ was < 0.15, indicating that using two reference genes is adequate and there is limited value added with additional reference genes.

### 2.5 Reffinder analysis

**Fig 4:**
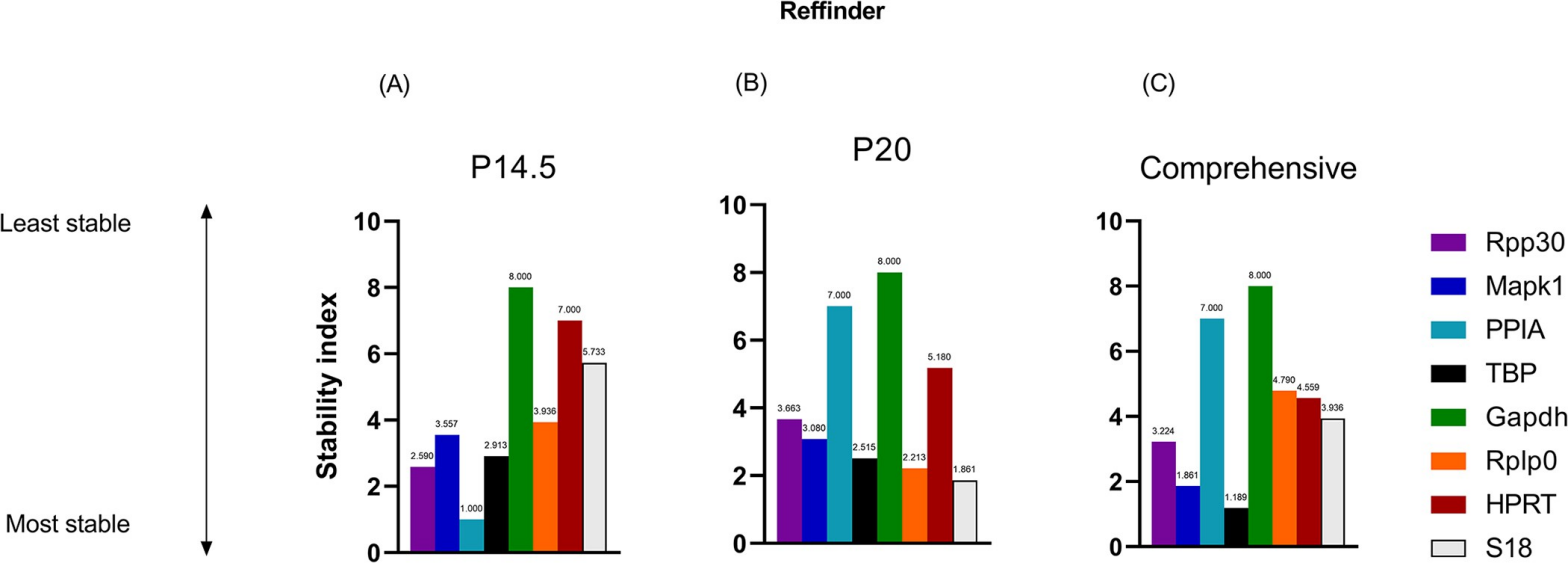
Stability ranking of 8 candidate reference genes by *RefFinder*. Lower stability index is associated with higher stability. (A) Early, highly avascular time point, P14.5. (B) Late time point where neovascularization is peaking, P20. (C) Comprehensive ranking of reference genes across both time points of P14.5.

To compare individual algorithms against the popular RefFinder algorithm, which creates a summary stability ranking from four individual algorithms, we analyzed the 8 candidate reference genes with this in silico prediction tool. Reference gene stability was ranked by the *RefFinder* program and the S.I. is expressed as a geometric mean. Higher stability of the candidate gene is correlated to lower geometric mean. Fig 4A displays the stability ranking at P14.5; Fig 4B displays the stability ranking at P20 and Fig 4C displays the comprehensive reference gene stability across both time points.

Across all groups and time points, TBP was predicted to be the most stable reference gene (Geometric mean (G.M.) 1.189) followed by MAPK1 (G.M. 1.861) and RPP30 (G.M. 3.224) (Fig 4C). At P14.5, PPIA (G.M. 1.000) and RPP30 (G.M. 2.590) were the most stable reference genes (Fig 4A). At P20, 18S (G.M. 1.861) and Rplp0 (G.M. 2.213) were the most stable genes (Fig 4B).

The least stable genes at P20 and across the comprehensive ranking of all groups and time points were GAPDH (G.M. 8) and PPIA (G.M. 7). At P14.5, GAPDH (G.M. 8) and HPRT (G.M.7) were the least stable reference genes.

Ranking of all genes and timepoints are summarized in Table 2.

**Table 2:** Summary of reference gene rankings by 3 primary algorithms and RefFinder, a summary tool, at each timepoint, P14.5, P20, and a comprehensive ranking of both timepoints.

	Timepoints	Normfinder	Bestkeeper	geNorm	Reffinder
1 (Most stable)	Comprehensive	RPP30	RPP30	RPP30	TBP
2		MAPK1	HPRT	MAPK1	MAPK1
3		PPIA	PPIA	TBP	RPP30
4		TBP	MAPK1	PPIA	18S
5		GAPDH	18S	Rplp0	HPRT
6		Rplp0	GAPDH	18S	Rplp0
7		HPRT	TBP	HPRT	PPIA
8 (Least stable)		18S	Rplp0	GAPDH	GAPDH
1 (Most stable)	P14.5	Rplp0	GAPDH	PPIA	PPIA
2		TBP	RPP30	RPP30	Rpp30
3		18S	HPRT	TBP	TBP
4		PPIA	18S	MAPK1	MAPK1
5		MAPK1	MAPK1	Rplp0	Rplp0
6		GAPDH	PPIA	18S	18S
7		RPP30	TBP	HPRT	HPRT
8 (Least stable)		HPRT	Rplp0	GAPDH	GAPDH
1 (Most stable)	P20	RPP30	RPP30	MAPK1	18S
2		MAPK1	PPIA	RPP30	Rplp0
3		TBP	MAPK1	GAPDH	TBP
4		PPIA	HPRT	TBP	MAPK1
5		GAPDH	GAPDH	PPIA	RPP30
6		18S	Rplp0	Rplp0	HPRT
7		HPRT	TBP	HPRT	PPIA
8 (Least stable)		Rplp0	18S	18S	GAPDH

### 2.6 Quantification of target gene compensated by reference genes

**Fig 5:**
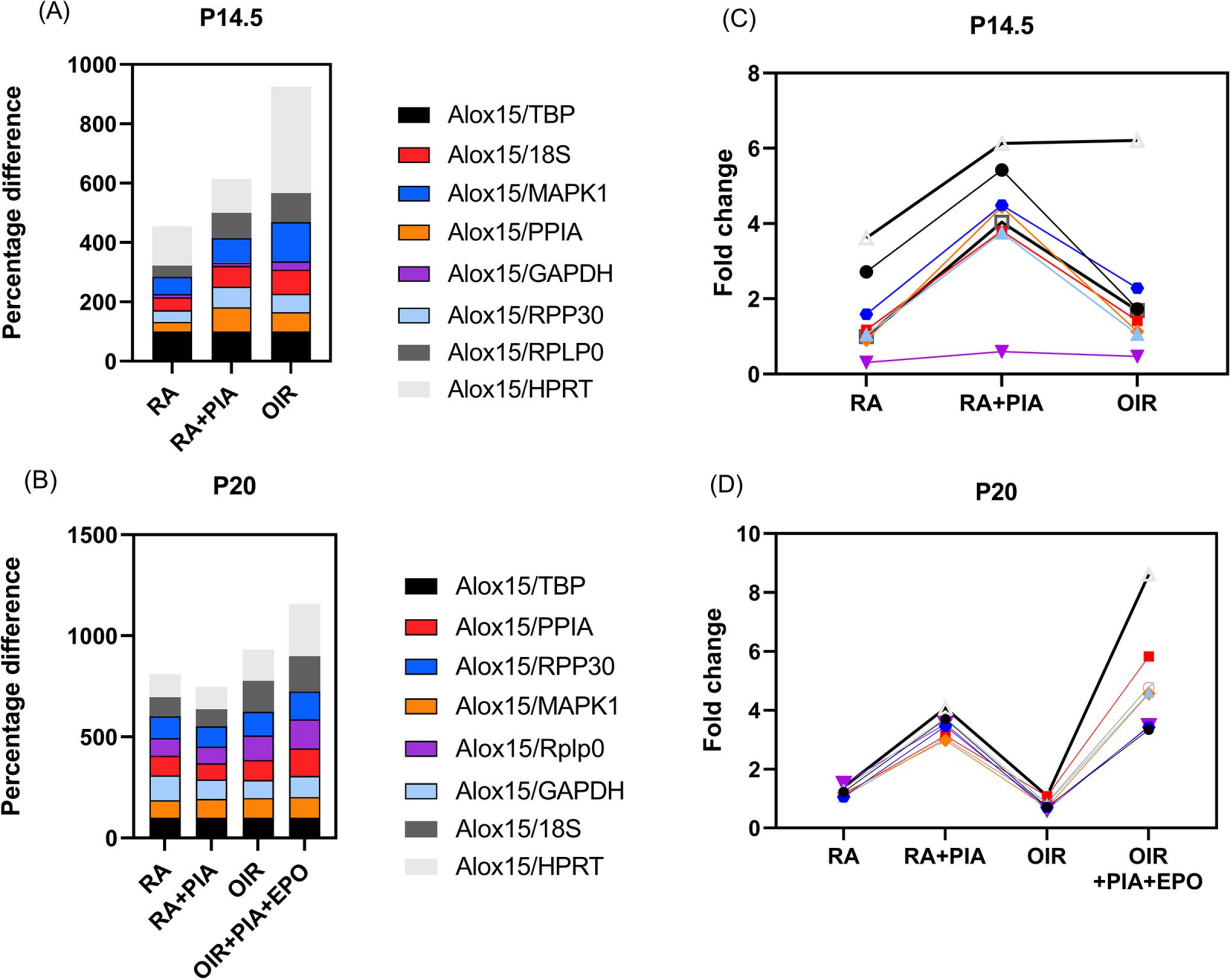
The percentage difference of expression level of Alox-15 in neonatal rat retinas normalized by different reference genes compared to TBP normalization at P14.5 by percentage (A), by fold change (C) and at P20 by percentage (B), and by fold change (D). Fold change was calculated by the 2^−ΔΔCt^ method. All results are expressed as relative amounts to the RA group of the respective time point.

Alox-15 mRNA expression was normalized to each reference gene at each individual time point, and the percentage difference was calculated by comparing to Alox15 mRNA expression level normalized to TBP. (Fig 5). At P14.5, fold change of Alox-15 normalized by two of the most highly stable genes across both time points, TBP (black) and MAPK1 (blue) resulted in similar values, 100% vs 82.68% respectively in RA+PIA group. Fold change normalized with GAPDH (purple), an unstable gene across both time points, led to a significantly lower value for all groups. There was about 70% difference in Alox-15 expression level between the a more stable reference gene, PPIA (orange), and a less stable gene, GAPDH in the RA+PIA group (Fig 5C and 5D).

In contrast, selection of reference genes did not cause significant disparity in all groups at P20. RA, RA plus PIA, and OIR groups had similar expression of Alox-15 regardless of the reference gene. The OIR plus PIA plus EPO group had higher Alox-15 expression when normalized to 18S, a more stable gene at this time point, although the variation was not significant compared to Alox-15 expression normalized by a less stable gene, GAPDH (*P* = 0.107) (Fig 5B).

## 3. Discussion

We have shown the stability of 8 commonly used reference genes across two time points that are pertinent to the developing retinal vasculature. TBP, MAPK1, and RPP30 are the most stable genes that are uninterrupted by anemia, OIR and EPO administration. We have demonstrated that normalization of RT-qPCR data with an unstable reference gene may lead to erroneous data conclusions. In this interest, identification of a stable reference gene that is not coregulated with the target gene is an absolute prerequisite.

Comprehensive stability ranking of the 8 candidate reference genes was determined through 3 validated algorithms (NormFinder, Bestkeeper, and geNorm) and compared to the comprehensive stability ranking predicted by *RefFinder*, an *in silico*, high throughput, popular, web-based, free program. This program has not been peer reviewed to our knowledge. It combines 4 programs (BestKeeper, GeNorm, NormFinder and comparative delta Ct) that use different methods to determine gene stability and gives a summary ranking as a geometric mean. It does not account for qPCR efficiency, so the user must only reliably use this tool when their own qPCR efficiency approximates 100%. Results from individual programs varied. Only in the comprehensive ranking, Normfinder, Bestkeeper, geNorm, all predicted RPP30 as the most stable reference gene, but at P14.5 and P20, no more than two algorithms resulted the same most stable reference genes.

Retinal development progresses rapidly during the neonatal phase, making it important to identify reference genes that are stable across multiple time points. Retinal development is highly impacted by OIR, necessitating testing of reference genes in both the avascular phase (P14.5 in this study) and the neovascular phase (P20). Furthermore, anemia and its treatment with EPO both alter iron homeostasis and impact cellular processes that may alter expression of genes typically thought to be stable in the retina [16]. In this study, TBP, MAPK1, and RPP30 were identified as stable genes across both time points between groups of all experimental conditions. GAPDH was most frequently identified as unstable and thus a poor candidate reference gene, and Rplp0 and 18S also received the least stable ranking in at least one algorithm so should not be used as single reference genes under these conditions.

The variability between algorithm results underscore the important point that in many experimental conditions, multiple reference genes should be used. In our study, GeNorm V_n_/V_n+1_ calculations determined that the use of 2 reference genes at each time point and comprehensively across time points is adequate. NormFinder suggested combinations of two reference genes in each analysis with the most stable S.I.: 18S and Tbp for use across both time points and at P14.5 and Mapk1 and Rpp30 at P20. In two of these three comparisons performed by NormFinder, the stability index of the combination of two genes was lower, indicating more stability, than the identified most stable single reference gene.

Due to differences in pathophysiology and cellular processes occurring at the two time points that may alter gene expression, analysis at each individual time point was also included. Table 2 shows the most and least stable genes at each time point, pertinent for experiments that only investigate one time point. The instability of reference genes between the two time points, being highly stable at P14.5 and highly unstable at P20, suggests that degree of transcription is affected in some of the most commonly used reference genes by the developmental stage of the retina.

We categorized the 8 tested genes based on their functional groups as metabolic enzymes (GAPDH, PPIA, MAPK1 and HPRT), ribosomal proteins (18S and RPLP0), and transcription factors (TBP and RPP30). Transcription factors appear to be more stable in a developing retina, although large-scale testing at different time points with more genes must be completed for confirmation. Specifically, RPP30 is ranked as the most stable reference gene 6 out of 9 times by Normfinder, Bestkeeper, and geNorm algorithms, Both TBP and RPP30 are ranked in the top most stable genes by Reffinder.

It is important to note that these experiments used whole retinal lysates, making the data generalizable only to other studies involving whole retina. The retina is a complex tissue composed of neural, vascular, and inflammation-regulating cell types. To determine candidate reference genes in a particular retinal cell type, further studies specific to cell type are required.

## 4. Conclusion

We demonstrated the importance of determining the stability of reference genes for RT-qPCR data normalization according to various developmental stages and experimental conditions. Depending on the developmental stage, a different reference gene or combination of two genes may be required. This study validates TBP, MAPK1 and RPP30 as the three most suitable reference genes for RT-qPCR analysis of rat retinal tissue with PIA, EPO, OIR and RA conditions at P14.5 and P20.

## 5. Methods

### 5.1 Animal procedures and retinal dissection

**Fig 6:**
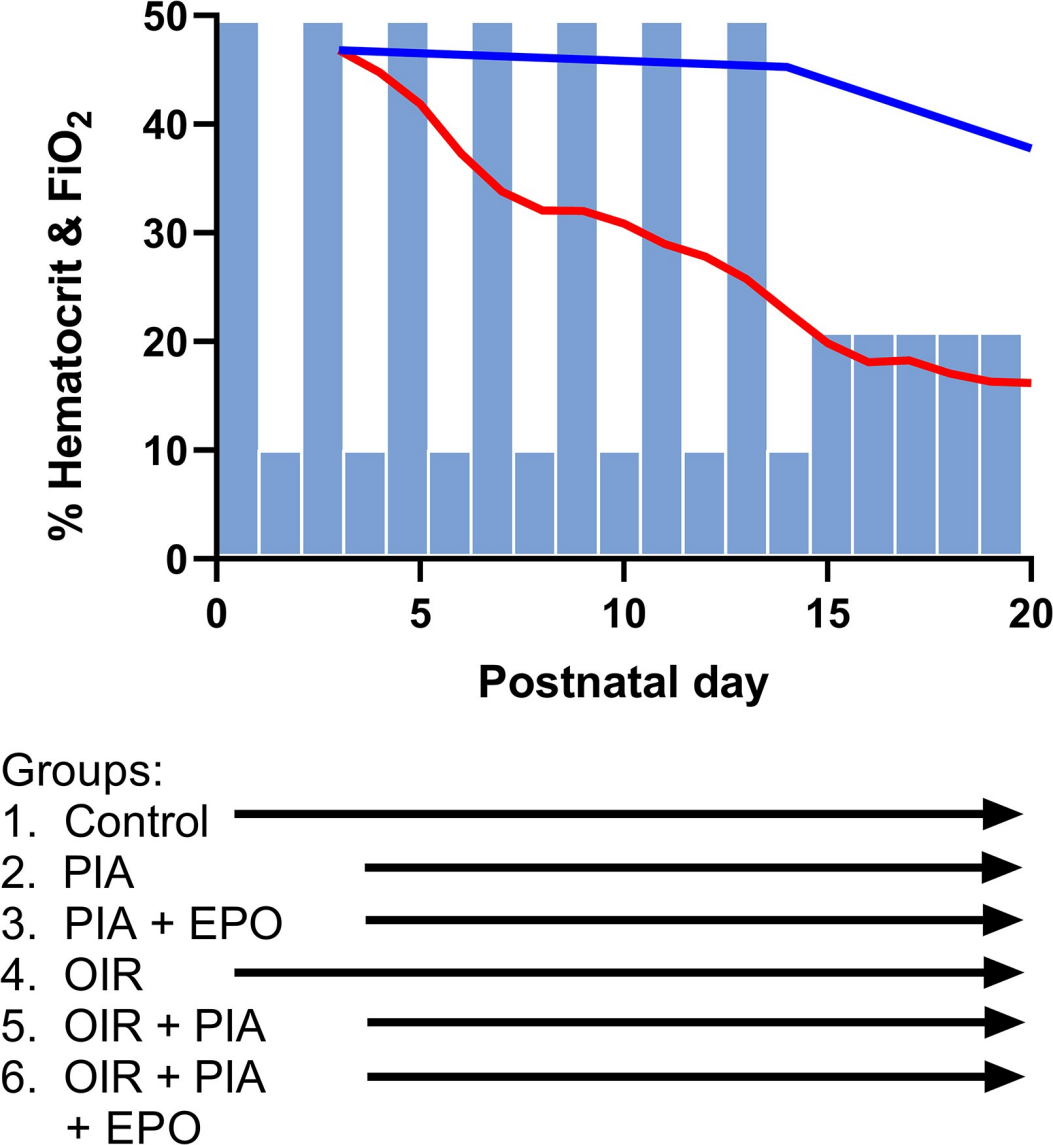
OIR and PIA animal models and experimental groups. In OIR groups, every 24 hours, the oxygen concentration was switched between 50% and 10% FiO_2_ until P14 when they were removed to room air (21% FiO_2_). Phlebotomy to induce PIA occurred from P3- P20 on hyperoxia days only, to prevent variation in oxygen concentrations during the highly sensitive hypoxia periods. Hematocrit levels of controls and PIA pups are shown. Bottom panel shows the experimental groups and timing of EPO administration.

All experiments were approved by the Institutional Animal Care and Use Committee at the University of Minnesota and comply with the National Institutes of Health guide for the care and use of Laboratory animals (NIH Publications No. 8023, revised 1978). Sprague Dawley timed pregnant rat dams, gestational day 15–16, were purchased from Charles River Laboratories (Wilmington, MA) and housed for one week prior to delivering the pups. Upon birth, the newborn rat pups were cross-fostered into litters of 18 pups to induce growth restriction according to the standard OIR model and assigned into groups with the following experimental conditions: room air (RA) control, phlebotomy-induced anemia (PIA), OIR and EPO (Fig 6). All animals were housed in a temperature and humidity controlled facility with time-cycled light (12 hours light, 12 hours dark). Pregnant and lactating dams had ad libitum access to standard rat chow and water.

RA animals were housed with a lactating dam in room air until euthanasia. OIR was induced using the Penn et al. 50/10 model [17]. Within 5 hours of birth, pups and dams were placed in a BioSpherix large animal chamber (BioSpherix, Redfield, New York), initially at 50% fraction inspired oxygen (FiO_2_). Every 24 hours, the oxygen concentration was cycled between 50% and 10% for 14 days. Oxygen concentrations were verified twice a day with external sensors. Small air ports in the chamber remained open throughout the experiments to allow for ventilation. Animals were then removed from the chamber to recover in room air until time of euthanasia.

The PIA model in these experiments was modified from the PIA model in mice, which successfully mimics the 50% reduction in hematocrit often occurring in preterm infants due to frequent phlebotomy [18]. PIA animals were phlebotomized 6ul/g from the facial vein once a day or twice daily to a target hematocrit of 15–20%. When animals were in the OIR chamber, phlebotomy occurred during the hyperoxia phase only to prevent FiO_2_ fluctuations during the sensitive hypoxia phase. As seen in Fig 6, pups reached their target hematocrit level at postnatal day (P) 14-P15. They were then phlebotomized once daily to maintain that degree of anemia. EPO was administered to half of the pups at 5000 units/kg through intraperitoneal injections from P10 to P20, alternating once or twice daily while in the chamber, during the hyperoxia phase from P10-P14, and twice daily thereafter until euthanasia. Dams and pups were monitored two times daily for the entirety of the experiments for signs of pain or distress. If a pup did not gain weight for two consecutive days, phlebotomy was not performed until weight gain was documented to alleviate suffering. Phlebotomy qualifies as Pain Class A according to the University of Minnesota Institutional Animal Care and Use Committee, so analgesia was not used for phlebotomy. This is consistent with practices for preterm infant phlebotomy. All pups remained in room air from P14 until euthanasia at P14.5 or P20. P14.5 represents the end of the hyperoxic-hypoxic injury and is a time of high retinal avascularity in the OIR model, mimicking the avascular Phase I of ROP. P20 is a time of peak neovascularization in the OIR model, mimicking the neovascular Phase II of ROP [17]. Rat pups were euthanized by carbon dioxide followed by confirmatory decapitation due to neonatal age. Immediately upon death, retinas were dissected and flash frozen in liquid nitrogen followed by -80C storage until further application.

### 5.2 Total RNA extraction and cDNA synthesis

Total RNA was extracted from individual whole retina using the RNaqueous RNA isolation kit (Ambion, TX) according to the manufacturer’s protocol. The quality and concentration were measured by Nanodrop ND-1000 (Thermo-Fisher Scientific, MA). Extracted total RNA samples were frozen and stored in -80C until cDNA synthesis. cDNA was synthesized using 1000ng of RNA in a High-Capacity cDNA reverse transcription kit with RNase inhibitor (Applied Biosystems, CA). cDNA was diluted to 1:10 with nuclease free water after synthesis and stored in -4C for short term or -80C for long term storage.

### 5.3 Quantitative PCR

The following four representative groups from each relevant time point were selected for RT-qPCR experiments to determine reference gene stability based on the inclusion of all 4 experimental conditions: RA control, RA plus PIA, OIR, and OIR plus PIA plus EPO. The RT-qPCR experiments were performed on 4–6 animals per group with equal representation of both female and male. Total reaction volume was 10ul: 4.5ul diluted cDNA, 0.5ul 20x commercial Taqman primer/probe (ThermoFisher,MA), and 5.5 ul Luna fast master mix (Cat. #M3004E, New England Biomed, MA). 8 commonly used housekeeping genes for conditions of OIR and anemia were selected from the literature- RPP30, TBP, PPIA, MAPK1, Rplp0, HPRT, 18S and GAPDH and were tested at both experimental endpoints (Table 1) [19–22]. Each sample was run in duplicate in a QuantStudio 3 qPCR machine (Applied Biosystems, MA). The RT-qPCR temperature profile included 95C for 1 min as an initial denaturation followed by 40 cycles of 95C for 15s and 60C for 30s.

Primer amplification efficiency of Alox-15 and each reference gene in each group was tested using slope analysis with a linear regression model. Relative standard curves were generated with 4 serial dilutions of cDNA (1:1, 1:10, 1:100, 1:1000). Each primer efficiency was calculated according to the equation: E  =  (10^(−1/slope)^ -1) x 100%.

### 5.4 In silico prediction of reference gene stability

We used Normfinder, geNorm, and Bestkeeper, three free, publically available algorithms validated to determine reference gene stability. We used these algorithms to predict stability rankings across all samples from both time points and then repeated the algorithms with data separated by P14.5 and P20 timepoints. The Normfinder algorithm was used as an excel add-in; available at https://moma.dk/normfinder-software. Inter and intragroup variations are calculated in Normfinder using a variance analysis-based approach [23]. GeNorm algorithm was accessed through qbase+ software, available at https://genorm.cmgg.be/. The gene expression stability measure, M, is calculated in geNorm as the average pairwise variation, V, for the gene with all other tested reference genes [15]. GeNorm also calculates V_n_/V_n+1_ for two sequential numbers of reference genes to determine whether the use of additional reference genes is required, with V_n_/V_n+1_ > 0.15 indicating an additional reference gene is required. Bestkeeper algorithm was also used as an excel add-in, available at https://www.gene-quantification.de/bestkeeper.html. Bestkeeper algorithm used standard deviation and variation deviation for stability ranking [13]. Average Ct values were used as the raw input data.

*RefFinder* (http://www.heartcure.com.au/reffinder/ and http://blooge.cn/RefFinder/), a free, online *in silico* prediction program was used to compare to the predicted stability of each tested gene in the above algorithms [24]. The program code is publicly available on the following website, https://github.com/fulxie/RefFinder. *RefFinder* integrates four programs, BestKeeper, NormFinder, geNorm, and comparative delta-Ct which all use different algorithms to rank stability of reference genes [13–15,25]. Based on the ranking of each program, appropriate weight was assigned to each gene and the final overall ranking was calculated by *RefFinder* as the geometric mean of each gene weight. *RefFinder* was run on all Ct values across both time points to generate a comprehensive ranking. It was again run on Ct values from the P14.5 endpoint groups only and P20 groups only to generate rankings specific to each time point.

### 5.5 Quantification of target gene expression

The expression level of the retinal enzyme, Arachidonate 15-lipoxygenase (Alox-15) was quantified in all four groups [26]. Alox-15 was selected from our RNA sequencing data due to its consistent alteration by the experimental conditions of OIR and PIA. Fold change of Alox-15 was calculated by the 2^−ΔΔCt^ method by each reference gene both for the comprehensive ranking across both time points and again at each individual time point [27]. All groups were normalized to the RA control group of their respective time point.

### 5.6 Statistical analysis

All groups were normally distributed with equal variance. Ct value outliers of the 8 candidate genes were identified by GraphPad Prism 8 (San Diego, CA) using the ROUT test (Q = 1%) before each ranking of stability was determined by *RefFinder*, *geNorm*, *Bestkeeper*, *and Normfinder*. Outliers of the target gene, Alox-15 were identified by the same methods after fold change was calculated. All outliers were excluded. The statistical significance of Alox-15 fold change compensated by each reference gene was determined by a two-tailed t-test using GraphPad Prism 8 or Microsoft Excel.

## Supporting information

S1 Raw data Ct values by group with and without outliers removed. (XLSX)

## Abbreviations

18S: Ribosomal protein 18S
Alox-15: Arachidonate 15-lipoxygenase
cDNA: Complimentary DNA
EPO: Erythropoietin
FiO_2_: Fraction of inspired oxygen
GAPDH: Glycealdehyde-3-phosphate dehydrogenase
GM: Geometric mean
HPRT: Hypoxanthine phosphoribosyl transferase
MAPK1: Mitogen-Activated Protein Kinase 1
mRNA: Messenger RNA
NICU: Neonatal intensive care unit
OIR: Oxygen induced retinopathy
P: Postnatal day
PIA: Phlebotomy induced anemia
PPIA: Peptidylprolyl Isomerase A
ROP: Retinopathy of Prematurity
Rplp0: Ribosomal protein, large, P0
RPP30: Ribonuclease P/MRP Subunit P30
RT-qPCR: Reverse-transcriptase quantitative polymerase chain reaction
SI: Stability Index
TBP: TATA box binding protein
